# CUL4B promotes prostate cancer progression by forming positive feedback loop with SOX4

**DOI:** 10.1038/s41389-019-0131-5

**Published:** 2019-03-14

**Authors:** Mei Qi, Jing Hu, Yanyi Cui, Meng Jiao, Tingting Feng, Xinjun Li, Yu Pang, Xinyi Chen, Ruixi Qin, Peng Su, Hui Zhang, Yan Wang, Yaoqin Gong, Bo Han

**Affiliations:** 1 The Key Laboratory of Experimental Teratology, Ministry of Education and Department of Pathology, Shandong University, School of Basic Medical Sciences, 250012 Jinan, China; 2 Department of Pathology, Shandong University Qi Lu Hospital, 250012 Jinan, China; 3 Department of Urology, The Fourth People’s Hospital of Jinan, 250031 Jinan, China; 4 Department of Pathology, Binzhou People’s Hospital, 256610 Binzhou, China; 5 Department of Pathology, Taian City Central Hospital, 271000 Taian, China; 6 Department of Pathology, The Affiliated Central Hospital of Qingdao University Medical College, 266042 Qingdao, China; 7 The Key Laboratory of Experimental Teratology, Ministry of Education and Department of Molecular Medicine and Genetics, Shandong University, School of Basic Medical Sciences, 250012 Jinan, China

## Abstract

How to distinguish indolent from aggressive disease remains a great challenge in prostate cancer (PCa) management. Cullin 4B (CUL4B) is a scaffold protein and exhibits oncogenic activity in a variety of human malignancies. In this study, we utilized PCa tissue specimens, cell lines and xenograft models to determine whether CUL4B contributes to PCa progression and metastasis. Here, we show that CUL4B expression highly correlates with the aggressiveness of PCa. CUL4B expression promotes proliferation, epithelial−mesenchymal transition, and metastatic potential of PCa cells, whereas CUL4B knockdown inhibits. Mechanically, CUL4B positively regulates SOX4, a key regulator in PCa, through epigenetic silencing of miR-204. In turn, SOX4 upregulates CUL4B expression through transcriptional activation, thereby fulfilling a positive feedback loop. Clinically, CUL4B+/SOX4+ defines a subset of PCa patients with poor prognosis. Bioinformatics analysis further reveals that Wnt/ß-catenin activation signature is enriched in CUL4B+/SOX4+ patient subgroup. Intriguingly, Wnt inhibitors significantly attenuates oncogenic capacities of CUL4B in vitro and in vivo. Together, our study identifies CUL4B as a key modulator of aggressive PCa by a positive feedback loop that interacts with SOX4. This regulatory circuit may have a crucial role in PCa progression.

## Introduction

Prostate cancer (PCa) is one of the most prevalent cancers for males worldwide^1^. The poor prognosis of PCa is mainly attributable to the high rate of tumor recurrence or metastasis, contributing to around 90% of cancer-related mortality^2^. Altered genes that play a driving role in PCa development and progression can often serve as specific diagnostic markers, criteria of molecular classification, and therefore potential therapeutic targets^3^. Thus far, several key molecular alterations and signaling pathways have been identified in PCa progression, including PTEN loss, TMPRSS2-ERG gene fusion, TP53 mutation, downregulation of NKX3-1, and SPOP mutation^4,5^.

Cullin 4B-Ring E3 ligases (CRL4B), assembled with Cullin 4B (CUL4B), DDB1, and ROC1 as the core components, participates in a broad variety of physiologically and developmentally controlled processes such as cell cycle progression, replication, and DNA damage response^6^. CRL4B catalyzes either polyubiquitination for proteasomal degradation or monoubiquitination at H2A (H2AK119ub1) for epigenetic modifications^7–9^. CUL4B was found to be overexpressed in multiple human cancers and possess potent oncogenic properties^10^. Recently, we and others have demonstrated that CUL4B repressed tumor suppressors that are highly important in solid malignancies, including P16, PTEN, Wnt antagonists and IGFBP3 at their promoters^8,11,12^.

The sex-determining region Y-box 4 (SOX4), a member of the C subgroup of SRY-related HMG box (SOX) transcription factor family, was reported to be overexpressed and correlated with poor clinical outcome in a variety of human malignancies^13^. Multiple studies have demonstrated that SOX4 plays a critical role in modulating the cellular proliferation, migration, invasion of tumor cells^14–18^. Mechanistically, SOX4 modulates key cellular regulators through direct transcriptional regulation, including the EGFR, EZH2, FOXA1 and NKX3.1, as well as at the posttranslational level through regulation of protein stability and interaction with specific cofactors, such as p53, syntenin-1 and β-catenin^13^. Our previous studies have reported SOX4 as an independent prognostic factor in Chinese PCa patients^15^. ERG may cooperate with SOX4 to promote epithelial−mesenchymal transition (EMT) in PCa progression^14^. Additionally, we and others have also demonstrated that aberrant SOX4 expression can also occur through microRNA (miRNA)-mediated regulation^19^.

In this study, we demonstrated that CUL4B expression is associated with aggressiveness of PCa. CUL4B positively modulates SOX4 protein expression by epigenetic silencing of miR-204. Reciprocally, SOX4 transcriptionally activates CUL4B expression through directly binding to its promoter region. Our findings suggest a positive feedback loop between CUL4B and SOX4 in regulating PCa progression. The CUL4B+/SOX4+ may define a subset of aggressive PCa with aberrant activation of Wnt/β-catenin signaling pathway.

## Results

### Overexpression of CUL4B is associated with poor prognosis in PCa

**Fig. 1:**
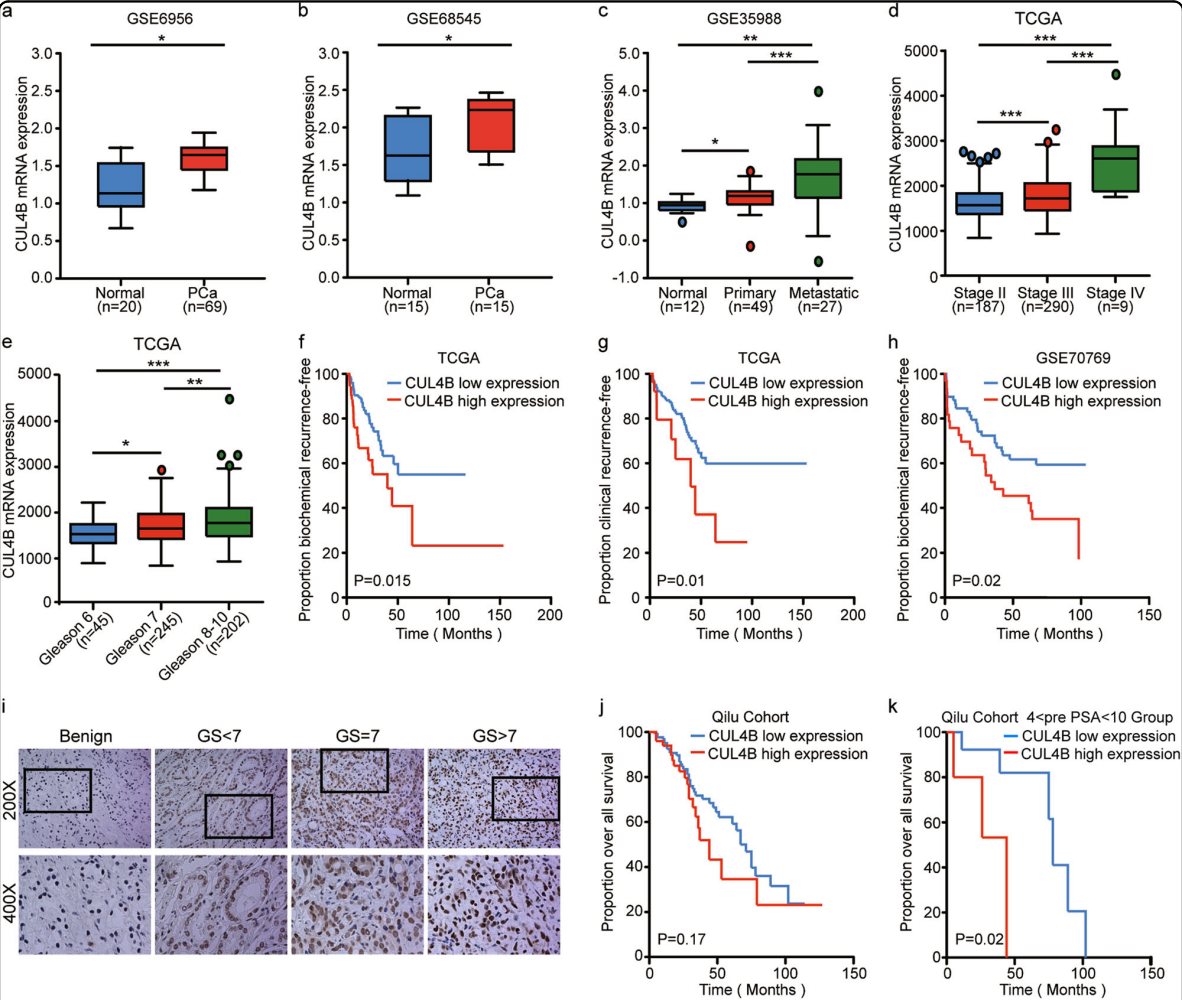
Overexpression of CUL4B predicts poor prognosis in prostate cancer (PCa). **a**, **b** The expression of CUL4B in PCa tissues compared with normal prostate samples in GSE6956 (**a**) and GSE68545 (**b**) dataset. **c** The expression of CUL4B in primary and metastatic PCa tissues compared with the matched normal prostate samples in GSE35988 dataset. **d**–**g** Analysis of CUL4B expression in TCGA dataset. **d** CUL4B levels in PCa subgroups with different TNM stages; **e** CUL4B levels in PCa subgroups with different Gleason scores; **f** the correlation between CUL4B expression and biochemical recurrence-free survival (*P* = 0.015, Log-rank test) and **g** clinical recurrence survival (*P* = 0.01, Log-rank test) in the TCGA cohort. **h** Kaplan–Meier survival analysis of microarray data from 94 PCa cases stratified by high and low CUL4B expression levels (*P* = 0.02, Log-rank test). **i**–**k** Analysis of CUL4B expression in Qilu cohort by IHC assay. **i** Representative IHC images of CUL4B expression of benign prostate tissue and PCa tissue with different Gleason score in Qilu cohort. **j** Kaplan–Meier survival analysis of PCa cases from Qilu cohort according to high and low CUL4B expression levels (*P* = 0.17, Log-rank test). **k** Kaplan–Meier survival analysis of PCa cases with Pre PSA level 4–10 ng/ml in Qilu cohort stratified by CUL4B levels (*P* = 0.01, Log-rank test). *P < 0.05, **P < 0.01, ***P < 0.001. PCa prostate cancer, TCGA The Cancer Genome Atlas, IHC immunohistochemistry

To investigate the clinicopathological significance of CUL4B expression of PCa patients, we first performed in silico analysis of CUL4B mRNA expression using published datasets. As shown in Fig. 1a, b, the CUL4B mRNA level was significantly higher in cancer tissues than that of benign prostatic tissues. We also found a significant gradual increase of CUL4B expression from benign prostatic tissue via primary PCa tissues to metastasis (GSE35988) (Fig. 1c). In TCGA cohort, CUL4B expression was correlated with Tumor, Lymph node and Metastasis (TNM) stage (Fig. 1d, none of the stage 4 patients received neoadjuvant treatment), and Gleason score (Fig. 1e). Notably, Kaplan−Meier survival analysis revealed that PCa patients with CUL4B overexpression had a faster progression to biochemical recurrence (*n* = 421, Fig. 1f) and clinical recurrence (*n* = 305, Fig. 1g). Similarly, association between CUL4B overexpression and biochemical recurrence was also identified in GSE70769 dataset (Fig. 1h).

Next, we investigated a cohort of 200 Chinese PCa cases from our hospital, Qilu cohort by IHC assay. As shown in Table 1, CUL4B overexpression in PCa was correlated with relatively higher Gleason score (*P* = 0.048). Representative IHC images of CUL4B in benign prostatic tissues as well as PCa cases with different Gleason score were shown in Fig. 1i. There was no significant association between CUL4B expression and age, pre-treatment prostate specific antigen (Pre PSA), pathological tumor stage or the presence of distant metastasis. Of note, PCa patients in our cohort with high expression of CUL4B displayed a relatively unfavorable overall survival (OS) (Fig. 1j; *n* = 200, Kaplan–Meier survival analysis), although this did not reach a statistical significance. However, we identified a significant association between the high CUL4B levels and poor OS in a subset of PCa patients with Pre PSA levels between 4 and 10 ng/ml (*n* = 20, Fig. 1k). Taken together, these results suggest that high levels of CUL4B are correlated with more aggressive behavior in PCa patients.

**Table 1:** Clinicopathological analysis of CUL4B expression in prostate cancer

Variables	CUL4B expression (%)		*P*
	Negative and weak	Moderate and strong	
Age (years)			
<65	24 (72.7)	9 (27.3)	0.969
≥65	122 (73.1)	45 (26.9)	
Pre PSA (ng/ml)^a^			
<4	13 (81.2)	3 (18.8)	0.816
4–10	15 (75.0)	5 (25.0)	
≥10	105 (73.9)	37 (26.1)	
Gleason score			
<7	23 (88.5)	3 (11.5)	0.048^b^
7	59 (76.7)	18 (23.3)	
>7	64 (65.9)	33 (35.1)	
Pathological tumor stage^a^			
≤pT2	105 (72.9)	39 (27.1)	0.749
≥pT3	31 (70.5)	13 (29.5)	
Distant metastasis^a^			
No	101 (74.3)	35 (25.7)	0.266
Yes	33 (66.0)	17 (34.0)	
Ki67			
<10%	125 (71.4)	50 (28.6)	0.185
≥10%	21 (84.0)	4 (16.0)	

^a^Values not available for all 200 cases

^b^*P* < 0.05

### CUL4B facilitates proliferation and invasion of PCa cells

**Fig. 2:**
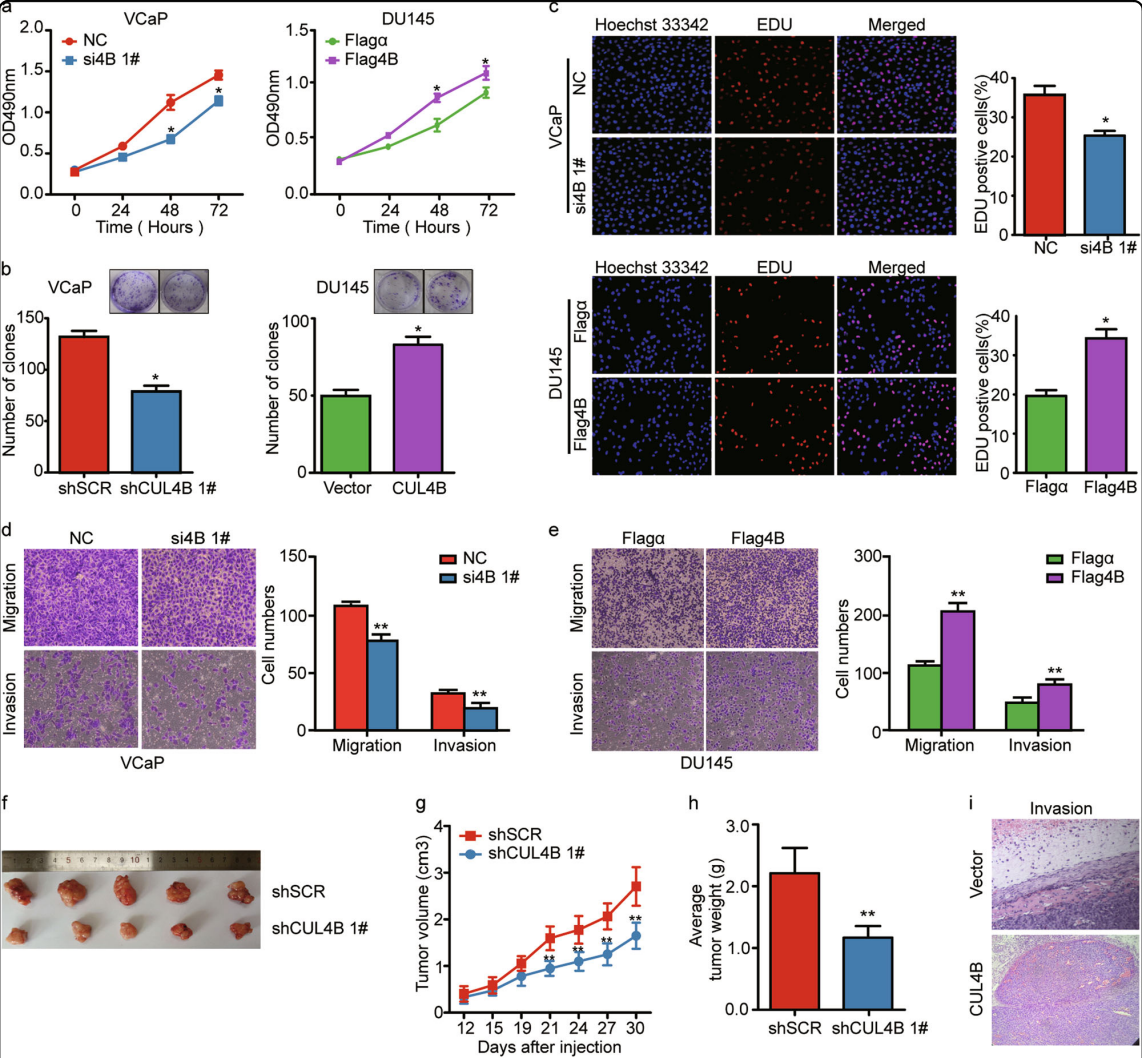
CUL4B promotes proliferation and invasion of PCa cells. **a** Cell viability as assessed by MTS assay at different time points, ranging from 0 to 72 h in VCaP and DU145 cells. si4B 1#/NC: CUL4B was knockdown in VCaP cells by transfection of 1# siRNA targeting CUL4B (si4B 1#) or a negative control siRNA (NC). Flag4B/Flagα: ectopic expression of CUL4B in DU145 cells by transfection of plasmid including Flag-tag and CUL4B gene sequence (Flag4B) or empty plasmid with Flag-tag only (Flagα). **b** Colony formation assay of indicated PCa cells with CUL4B ablation or expression results after knocking down or overexpressing CUL4B in VCaP and DU145 cells. Quantitative analysis of colony numbers is shown in the right panel. For colony formation assays, colonies containing more than 50 cells were counted and plotted. Left panel: VCaP cells stably transfected 1# shRNA targeting CUL4B (shCUL4B 1#) or negative control (shSCR) by virus. Right panel: DU145 cells stably transfected plasmid containing CUL4B gene sequence (CUL4B) or empty plasmid (Vector) by virus. VCaP and DU145 cells stably expressing corresponding shRNA or plasmids were maintained in culture media for 14 days prior to being stained with crystal violet. **c** Representative photographs of EdU assays of VCaP cells transfected with si4B 1#/NC or DU145 cells transfected with Flag4B/Flagα as indicated. **d**, **e** The effect of CUL4B on cell migration/invasion evaluated by transwell migration and matrigel invasion assays. **d** Transwell assay of VCaP cells transiently transfected with si4B 1#/NC. **e** Transwell assay of DU145 cells transiently transfected with Flag4B/Flagα. Left panel: Representative images of cell migration and invasion. Right panel: Quantitative results of migration and invasion assays from triplicate experiments. In each experiment, cells were counted in five random fields of each filter under a microscope using a ×40 magnification. **f**–**h** Effect of CUL4B on turmorigenesis in vivo evaluated with xenografts model. VCaP cells with stable expression of shSCR/shCUL4B 1# subcutaneously injected into nude mice. Tumors were measured every 3 days using a vernier calliper, and the volume was calculated according to the formula: 1/6 × length × width^2^. Representative images of xenograft tumors (**f**), growth curves of tumors (**g**), and the average weight of tumor mass in each group (**h**) were shown. **i** Representative H&E images of xenograft tumor well encapsulated in the Vector control group vs. xenograft with capsule invasion in the CUL4B group. DU145 cells with stable expression of Vector/CUL4B subcutaneously injected into nude mice. HE stain was performed to each tumor at harvest time. Each bar represents the mean ± SD of three independent experiments. *P < 0.05, **P < 0.01. PCa prostate cancer

We examined CUL4B expression in a series of PCa cell lines (DU145, LNCaP, 22RV1, VCaP, PC3 and immortalized nontumorigenic prostate epithelial cell line, RWPE) (Figure S1a), the expression of CUL4B was higher in PCa cell lines than that in RWPE cell line. We then performed gain- and loss-of-function analyses in VCaP and DU145 cells transfected with CUL4B knockdown or overexpression, accordingly (Figure S1b and S1c). We found that CUL4B downregulation significantly decreased cell proliferation whereas CUL4B overexpression promoted cell growth, as measured by MTS assay (Fig. 2a, S1d and S1e) and colony formation assay (Fig. 2b and S1f). These results were further confirmed by EdU assay (Fig. 2c and S1g). Invasion and metastasis are major hallmarks of cancer cells. In the present study, we demonstrated that knockdown of CUL4B by siRNA significantly suppresses migration and invasion in VCaP cells, whereas overexpressing CUL4B has the opposite effect in both VCaP and DU145 cells (Fig. 2d, e and S1h).

Furthermore, we investigated the role of CUL4B in PCa in vivo. Compared to a negative control (ShSCR), shRNA-mediated knockdown of CUL4B (shCULB) significantly inhibited the growth of VCaP tumor xenografts (Fig. 2f–h). Ectopic expression of CUL4B (CUL4B) significantly promoted the growth of DU145 tumor xenografts (Figure S1i and S1j), compared to a control group (Vector). In addition, the subcutaneous tumors with CUL4B overexpression displayed signs of outside-invasion of the capsule, whereas the xenograft tumors of the Vector group were well encapsulated without invasion (Fig. 2i). In all, these results revealed that CUL4B has a strong oncogenic ability in PCa.

### CUL4B promotes epithelial−mesenchymal transition in PCa

**Fig. 3:**
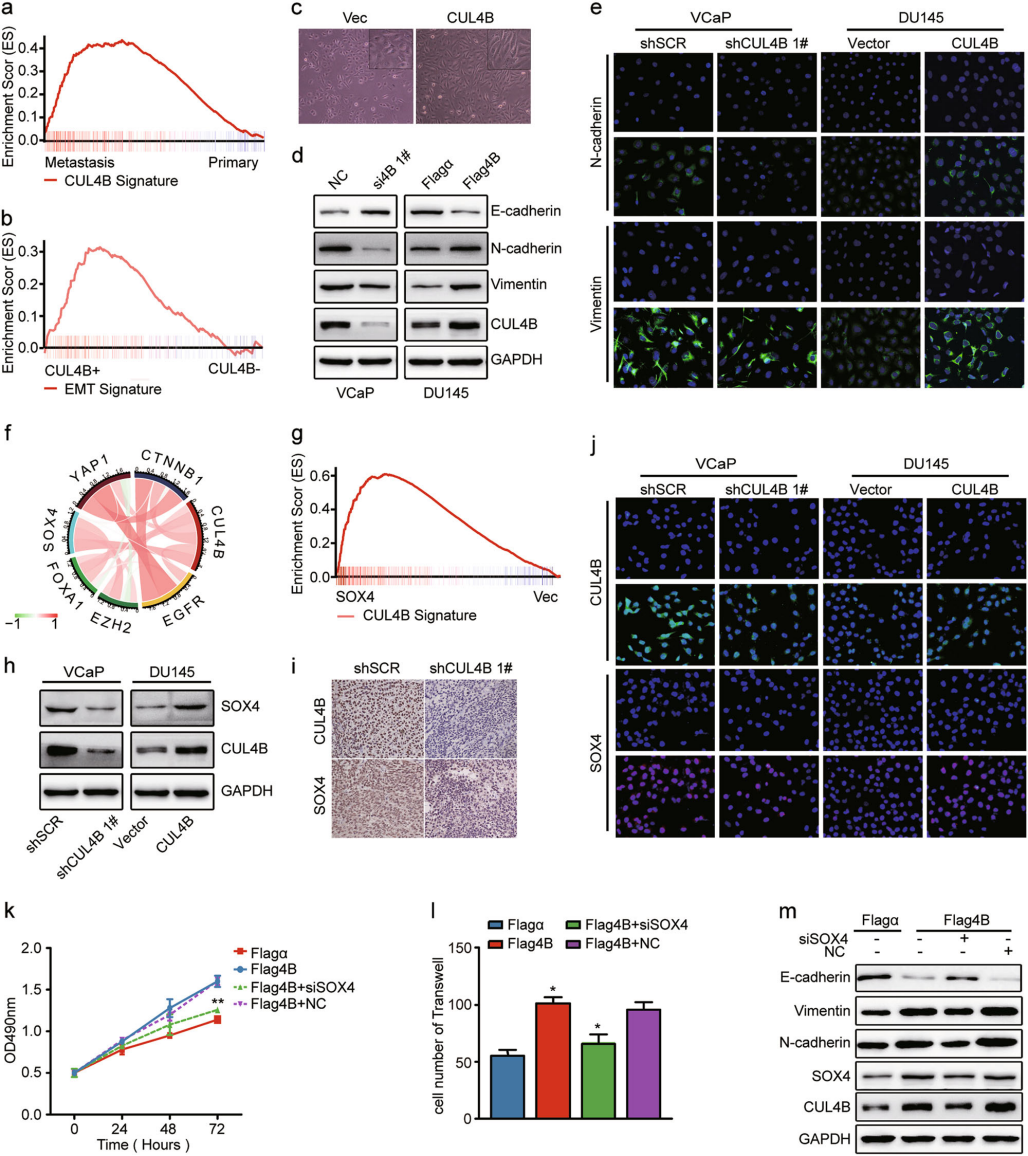
CUL4B promotes EMT in PCa through SOX4. **a** Gene set enrichment analysis (GSEA) of CUL4B coexpressed signature (TCGA) in mRNA microarray of metastasis PCa (GSE32269). Enrichment scores (ES) were shown on the *y*-axis. *x*-axis bars represent individual genes of the indicated gene sets. ES = 0.43, *P* < 0.001, FDR *q* < 0.001. **b** EMT gene signatures in GSE35988 grouped by CUL4B expression with GSEA. ES = 0.29, *P* = 0.01, FDR *q* = 0.14. **c** Representative morphologic changes of VCaP cells transfected with Vector/CUL4B (phase-contrast illustration). **d** Western blot analysis of E-cadherin, N-cadherin, Vimentin, and CUL4B expression. VCaP cells were transiently transfected with siRNA targeting CUL4B (si4B 1#) or negative control (NC) and DU145 with plasmid expressing CUL4B (Flag4B) or empty plasmid (Flagα). GAPDH was used as a loading control. **e** Immunofluorescence analysis of localization and expression levels of EMT markers (N-cadherin and Vimentin) in indicated PCa cells. **f** Circos plot displaying the interconnectivity among genes related to PCa EMT. The thickness and color of the ribbons correlate to the correlation of genes expression in TCGA dataset (Supplementary Table 2). **g** GSEA of CUL4B coexpressed signature (TCGA) in mRNA microarray of PCa cells ectopic expressing SOX4 (GSE11914). ES = 0.43, *P* < 0.001, FDR *q* < 0.001. **h** Western blot analysis of CUL4B and SOX4 expression in PCa cells with CUL4B knockdown or overexpression. **i** Representative IHC images of CUL4B and SOX4 expression in xenograft tumors derived from VCaP cells. **j** Immunofluorescence staining analysis of CUL4B and SOX4 expression in PCa cells. **k**–**m** Effect of SOX4 in oncogenic function of CUL4B. DU145 cell were transiently transfected with empty plasmid (Flagα), CUL4B expression plasmid (Flag4B), control siRNA (NC), or SOX4 siRNA (siSOX4) as indicated. Cells were subjected to MTS assay (**k**), transwell migration (**l**) and western blot assay (**m**). *P < 0.05, **P < 0.01. EMT epithelial−mesenchymal transition, PCa prostate cancer, TCGA The Cancer Genome Atlas

Gene set enrichment analysis (GSEA) of PCa microarray data (GSE32269 and TCGA) revealed that CUL4B expression was significantly associated with metastasis signature (Fig. 3a) and EMT signature (Fig. 3b), respectively. The ranking metric scores and leading edge subset of EMT signature were listed in Supplementary Table 1. Activation of an EMT program is coupled with phenotypic plasticity and genetic alteration^20^. Here we showed PCa cells with CUL4B expression acquired a dispersed, spindle-shaped morphology (Fig. 3c). As assayed by western blot and real-time PCR, the depletion of CUL4B resulted in an increase in E-cadherin protein expression but decreases in N-cadherin and Vimentin expression in VCaP cells. By contrast, CUL4B overexpression had the opposite effect on EMT markers (Fig. 3d, S2a, S2b and S2c). These results were further confirmed by immunofluorescence (Fig. 3e and S2d).

### CUL4B regulates EMT in a SOX4-mediated manner

As shown in Fig. 3f, TCGA data revealed a positive correlation between the expression of CUL4B and EMT-related genes in PCa, FOXA1, EZH2, CTNNB1 YAP1, SOX4, and EGFR. Interestingly, GSEA revealed that CUL4B-induced genes were significantly enriched for upregulation upon SOX4 overexpression (Fig. 3g). Knockdown of CUL4B in VCaP cells resulted in marked decrease in SOX4 protein levels, rather than mRNA levels. In contrast, SOX4 protein levels were significantly increased in DU145 cells with CUL4B overexpression (Fig. 3h). Moreover, subcutaneous xenografts derived from VCaP cells showed decreased SOX4 expression in shCUL4B xenograft tissues by IHC (Fig. 3i). Consistent with these data, expression of CUL4B and SOX4 was verified by immunofluorescence (Fig. 3j).

As shown in Fig. 3k, l, CUL4B overexpression alone significantly induced proliferation and invasion of VCaP cell, while SOX4 knockdown partially blocked CUL4B-induced cell proliferation and invasion. Further analysis by western blot showed that CUL4B overexpression significantly decreased the expression of epithelial marker (E-cadherin) but increased mesenchymal markers (N-cadherin and Vimentin). More importantly, these changes were partially reversed by SOX4 knockdown (Fig. 3m). Therefore, CUL4B induced proliferation and invasion through SOX4 regulation in PCa cells.

### CUL4B induces SOX4 expression through modulating miR-204 level

**Fig. 4:**
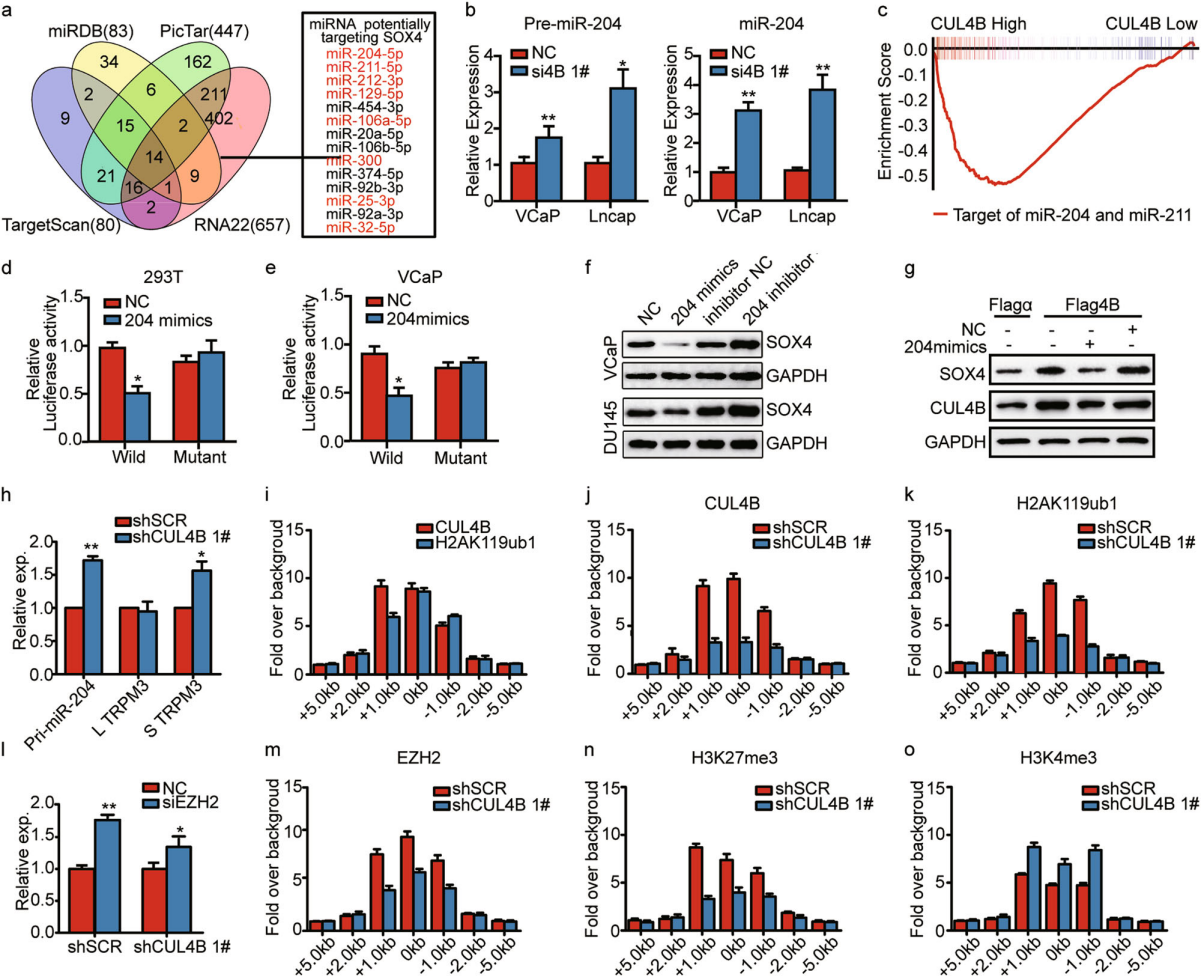
CUL4B induces SOX4 expression by modulating miR-204 level. **a** Venn diagram showing candidate miRNAs targeting SOX4. SOX4 3′-UTR region was used for analysis of miRNA-binding sites by using four different bioinformatics tools including TargetScan, miRDB, PicTar and RNA22. **b** Relative expression of precursor miR-204 (pre-miR-204) and mature miR-204 (miR-204) in PCa cells. VCaP or LNCaP cells transiently transfected with control siRNA (NC), or CUL4B siRNA (si4B 1#) were subjected to real-time PCR. **c** GSEA of miR-204 signature in TCGA dataset grouped by CUL4B expression. Enrichment scores (ES) were shown on the *y*-axis. *x*-axis bars represent individual genes of the indicated gene sets. ES = −0.52, *P* < 0.001, FDR *q* < 0.001. **d**, **e** Luciferase reporter assay of HEK293T (**d**) and VCaP cells (**e**). 293T and VCaP cells were transiently transfected with wild-type construct of pmirGLO-SOX4 3′-UTR (Wild), mutant construct of pmirGLO-SOX4 3′-UTR (Mutant), control RNA (NC) or miR-204 mimics (204 mimics) as indicated. **f** Western blot analysis of SOX4 expression in VCaP and DU145 cells transfected with miR-204 mimics or inhibitor and relative control RNA. **g** Western blot analysis of SOX4 and CUL4B expression in DU145 cells. DU145 cells were transient transfected with empty plasmid (Flagα), CUL4B expression plasmid (Flag4B), control RNA (NC), or miR-204 mimics (204 mimics) as indicated. prior to western blot assay. **h** Real-time PCR of indicated transcripts of TRPM3 in VCaP cells. VCaP cells with stable expression of shSCR/shCUL4B were used to exam the expression of indicated transcripts of TRPM3 with specific primes. **i** ChIP-qPCR analysis of recruitment of CUL4B and H2AK119ub1 at promoters of miR-204 in VCaP cells. Purified rabbit IgG was used as a negative control for background enrichment signal. ChIP enrichments were presented as fold over background signal. Error bars represent mean ± SD of three independent experiments. **j**, **k** ChIP-qPCR analysis of recruitment of CUL4B (**j**) and H2AK119ub1 (**k**) at promoters of miR-204 in VCaP after transfection with control shRNA(shSCR) or shRNAs targeting CUL4B (shCUL4B 1#). Error bars represent mean ± SD of three independent experiments. **l** Expression of miR-204 was determined by real-time PCR from VCaP cells. CUL4B-knockdown and control VCaP cells transiently transfected with control siRNA (NC), or EZH2 siRNA (siEZH2) as indicated before real-time PCR assay. **m**–**o** ChIP-qPCR analysis of recruitment of EZH2 (**m**), H3K27me3 (**n**), and H3K4me3 (**o**) at promoters of miR-204 in CUL4B-knockdown and control VCaP cells. Error bars represent mean ± SD of three independent experiments. *P < 0.05, **P < 0.01. PCa prostate cancer, TCGA The Cancer Genome Atlas

As shown in Figure S3, no statistical alteration of SOX4 mRNA level was identified by dysregulation of CUL4B. Further experiment indicated that CUL4B was not involved in the proteasomal degradation of SOX4 (data not shown). We therefore hypothesized that CUL4B might regulate SOX4 expression via posttranscriptional level and miRNA might be involved. Using online tools including TargetScan, miRDB, PicTar and RNA22, and combined with previous studies, we integrated eight putative tumor-suppressor miRNAs targeting SOX4: miR-204, miR-211, miR-212, miR-129, miR-106a, miR-300, miR-25, and miR-32 (Fig. 4a). To verify the interaction of CUL4B with candidate miRNAs, we first examined the expression of pri-miRNAs in VCaP cells with CUL4B knockdown or overexpression. Among them, miR-204 was found to be the one that was the most significantly changed (Fig. 4b, S4a and S4b). In addition, GSEA revealed a significantly reverse correlation between CUL4B expression and miR-204 levels (Fig. 4c). We therefore chose miR-204 for further investigation.

As shown in Fig. 4d, e, luciferase reporter assay illustrated that while miR-204 overexpression in HEK293T and VCaP cells significantly inhibited the relative luciferase activity of the wild-type construct of pmirGLO-SOX4 3′-UTR, whereas the activity of the mutant construct of pmirGLO-SOX4 3′-UTR was only marginally repressed. Furthermore, western blot analysis confirmed that SOX4 protein level was remarkably repressed by miR-204 mimics yet increased by miR-204 inhibitor (Fig. 4f). In all, our data demonstrated that SOX4 is a target of miR-204 in PCa.

To validate the role of miR-204 in the induction of SOX4 by CUL4B, we transiently transfected VCaP cells with Flag4B/Flagα in combination with NC/miR-204 mimics. Western blot analysis confirmed that reconstitution of miR-204 in these cells indeed greatly suppressed SOX4 level, thereby reversing its induction by CUL4B (Fig. 4g). Our data thus supported miR-204 as an important mediator of CUL4B-induced SOX4 expression.

### CUL4B recruits PRC2 complex to miR-204 promoter and inhibits its transcription

Referred to UCSC and Olga’s study^21^, miR-204 encodes within intron 6 of TRPM3, and shares the transcription start site (TSS) with a short transcript, ENST00000361823, rather than the long transcript ENST00000377110, encoding the full-length protein (Figure S5a). The expression of TRPM3 and miR-204 showed significant correlation in PCa according to TCGA data (Figure S5b). As shown in Fig. 4h, CUL4B-induced regulation of miR-204 correlated with expression of short transcripts rather than the long transcript of TRPM3, suggesting the regulation in transcriptional level. Next, we examined CUL4B enrichment and consequential monoubiquitination of histone H2A in the promoter region of miR-204. ChIP assay in VCaP cells with antibodies against CUL4B, H2AK119ub1 or control immunoglobulin G (lgG) indicated that CUL4B and H2AK119ub1 co-occupied the promoter of miR-204 (Fig. 4i and S5c). Consistent with these results, shCUL4B significantly reduced the levels of CUL4B binding to the promoters as well as those of H2AK119ub1 (Fig. 4j, k). These results indicate that CUL4B repressed miR-204 expression by directly bind to the promoter region.

PCa patients with high CUL4A expression can benefit from thalidomide, the Cereblon (CRBN) inhibitor. This is mainly due to function of CRBN as the substrate receptor in E3 ubiquitin ligase complex CRL4A^CRBN^ ^22^. Next, we investigated whether CRBN is the substrate receptor of CUL4B inducing monoubiquitination of H2AK119 by thalidomide treatment. As shown in Figure S5d, thalidomide failed to reverse repression of miR-204 by CUL4B. VEGFA was chosen to be the positive control for thalidomide treatment^23^.

It was previously reported that CUL4B could act in concert with PRC2 to repress target genes^8^. It is well documented that EZH2 is the catalytic component in PRC2. We therefore tested whether CUL4B-repressed transcription of miR-204 was EZH2 dependent or not. As shown in Fig. 4l, inhibition of EZH2 increased levels of miR-204 but in a CUL4B-dependent manner. ChIP assay revealed that the promoter regions of miR-204 were highly enriched for EZH2 and H3K27me3, with occupancy sites overlapping with those of CUL4B (Fig. 4m, n). In addition, knockdown of CUL4B markedly reduced the enrichment of EZH2, as well as the associated H3K27me3 mark in these regions (Fig. 4m, n). Trimethylation of lysine 4 on histone H3 (H3K4me3) is a universal chromatin modification at the transcription start site of active genes^24^. The increased enrichment of H3K4me3 on miR-204 promoter region after CUL4B inhibition reflected increased transcription of pri-miR-204 (Fig. 4o). In all, our data supported that CUL4B epigenetically silenced miR-204 transcription through recruitment of PRC2 complex.

### SOX4 reinforces CULB by forming positive feedback loop

**Fig. 5:**
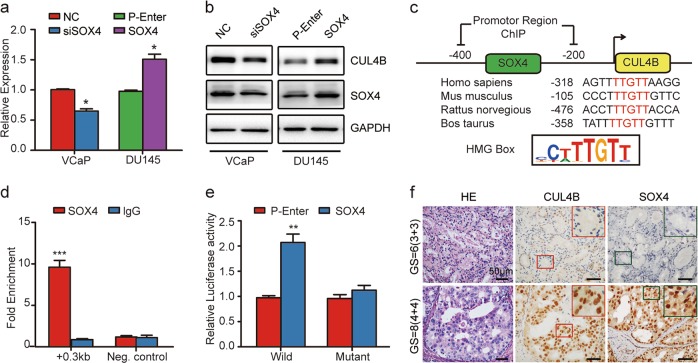
SOX4 reinforces CUL4B by forming feedback loop. **a**, **b** CUL4B mRNA (**a**) and protein (**b**) expression determined after SOX4 transient knockdown or overexpression. VCaP cells transiently transfected with control siRNA (NC), or SOX4 siRNA (siSOX4), and DU145 transiently transfected with empty plasmid (P-Enter), SOX4 expression plasmid (SOX4) were subjected to real-time PCR and western blot assay. **c** Schematic representation of SOX4 binding sites, HMG box, in the CUL4B promoter. The loci and sequence of HMG box among different species were shown in the middle. **d** ChIP-qPCR analysis of recruitment of SOX4 at promoter region of CUL4B in VCaP cells. Purified rabbit IgG was used as a negative control. ChIP enrichments were presented as fold over negative control. Error bars represent mean ± SD of three independent experiments. **e** Luciferase reporter assay was used to determine whether CUL4B was a direct target of SOX4 in HEK293T cells. 293T cells were transiently transfected with wild-type construct of pGL3-CUL4B HMG box (Wild), mutant construct of pGL3-CUL4B HMG box (Mutant), empty plasmid (P-Enter), SOX4 expression plasmid (SOX4) as indicated. *P < 0.05, **P < 0.01, ***P < 0.001. **f** Representative images of HE and IHC staining of CUL4B and SOX4 in Qilu cohort with different Gleason score. GS Gleason Score, IHC immunohistochemistry

SOX4 is a transcription factor that regulates many genes by binding to their promoters. This prompted us to further investigate whether SOX4 could regulate CUL4B expression at mRNA level. As assayed by real-time PCR and western blot, the expression of CUL4B showed consistent pattern with SOX4 in VCaP and DU145 cells (Fig. 5a, b). According to online tools including JASPAR, Genomatrix and PROMO, we obtained HMG box, SOX4 binding site, in a region approximately 0.3 kb upstream from the TSS of the *CUL4B* gene (Fig. 5c). ChIP assay revealed that antibody against SOX4 efficiently immunoprecipitated this region in VCaP cells (Fig. 5d). To investigate whether SOX4 activates the CUL4B promoter, a luciferase reporter assay was performed in 293T cells transfected with pGL3-CUL4B promoter vector containing wild-type HMG (Wild) or mutant HMG (Mutant). As shown in Fig. 5e, SOX4 increased the promoter activity in cells transfected with wild type, but not in cells with mutant. We next examined whether CUL4B and SOX4 is physiologically relevant in human PCa cases. As shown in Fig. 5f, the protein levels of CUL4B and SOX4 by IHC were much higher in PCa cases with Gleason score 8–10 than those with Gleason score 6–7. Notably, CUL4B overexpression was significantly associated with SOX4 overexpression by IHC (*P* = 0.001) in our cohort. These data indicate a reciprocal loop of CUL4B and SOX4 in PCa.

### CUL4B+/SOX4+ defines a subset of aggressive PCa with poor prognosis

**Fig. 6:**
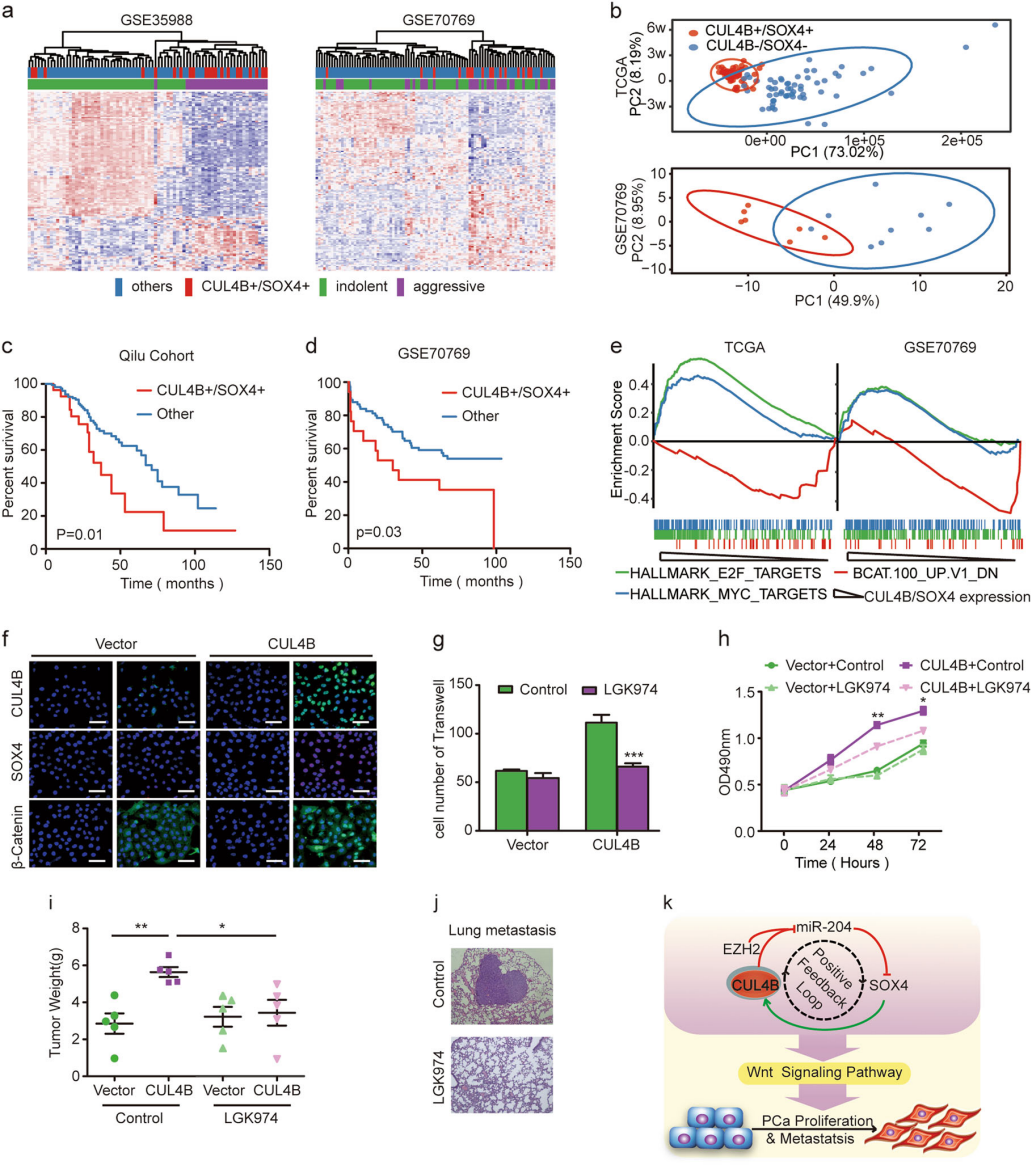
CUL4B+/SOX4+ subgroup with unfavorable outcome and aberrant activation of Wnt/β-catenin signaling pathway. **a** Unsupervised clustering analysis of GSE35988 (left) and GSE70769 (right) datasets based on differentially expressed genes (DEGs) of indolent and aggressive PCa. Patients statue were shown in the annotation column. Patients were annotated according to CUL4B and SOX4 expression or PCa risk assessment. Red: patients with concurrent high CUL4B and SOX4 expression (CUL4B+/SOX4+); Blue: other patients (other); Green: patients with indolent PCa (indolent); Purple: Patients with aggressive PCa (aggressive). **b** Principle component analysis (PCA) of unique differentially expressed genes between CUL4B+/SOX4+ and CUL4B−/SOX4− patients in TCGA and GSE70769 datasets. Each plot represents a patient. **c**, **d** The correlation between CUL4B+/SOX4+ subgroup and overall survival in the Qilu PCa cohort (**c**) (*n* = 200, *P* = 0.01, Kaplan−Meier survival analysis, Log-rank test) and GSE70769 (**d**) (*n* = 94, *P* = 0.03, Kaplan−Meier survival analysis, Log-rank test). **e** GSEA of statistically significant overlapping gene signatures in TCGA (left panel) and GSE70769 (right panel) datasets. Green for E2F activated signature, blue for C-MYC activated signature, and red for signature containing genes downregulated upon β-catenin activation (*P* < 0.05 and FDR < 0.2). **f** Immunofluorescence analysis of the localization and expression levels of SOX4 and β-catenin in DU145 cells. DU145 cells stable transfected with empty plasmid (Vector) or CUL4B expressed plasmid (CUL4B) were subjected to immunofluorescence analysis. Scale bar, 25 μm. **g**, **h** Cell migratory (**g**) and proliferation (**h**) capacities of DU145 cells. CUL4B-overexpressing and control DU145 cells were treated with LGK974 (10uM) or control before transwell migration (**g**) and MTS assay (**h**). **i**, **j** DU145 cells with stable expression of Vector/CUL4B subcutaneously injected into nude mice. The experiments were separated into four groups: Vector + PBS, CUL4B + PBS, Vector + LGK974, CUL4B + LGK974, *n* = 5/group). **i** The weight of tumors formed at harvest time. **j** Representative images of pulmonary metastatic loci. **k** Schematic model of positive feedback loop between CUL4B and SOX4 in regulating PCa progression. *P < 0.05, **P < 0.01, ***P < 0.001. PCa prostate cancer, TCGA The Cancer Genome Atlas

We next performed a cross-platform analysis to stratify high-risk PCa patients (aggressive disease) from low-risk patients (indolent disease)^25^. In GSE35988 and GSE70769 PCa datasets, samples were divided into patients with increased levels of both CUL4B+/SOX4+ and others (Fig. 6a). Patients with concurrent high CUL4B and SOX4 expression (CUL4B+/SOX4+) tightly cluster apart from other patients and congruent with aggressive PCa subgroup. As shown in Figure S6a and Fig. 6b, unsupervised hierarchical clustering and principal component analysis (PCA) demonstrated that CUL4B+/SOX4+ PCa patients displayed a different set of differentially expressed genes (DEG) compared to CUL4B-/SOX4- PCa patients. Notably, CUL4B+/SOX4+ patients had a worse overall survival rate than the others, including CUL4B−/SOX4− patients (Fig. 6c and Figure S6b). This feature was also identified in the GSE70769 dataset (Fig. 6d). These data suggested that CUL4B+/SOX4+ defines a subset of aggressive PCa with poor prognosis.

Although androgen-deprivation treatment is the mainstay treatment for advanced PCa, it eventually fails and patients invariably progress to castration-resistant prostate cancer (CRPC). Our previous study demonstrated that SOX4 expression is upregulated in CRPC tumors, as well as C4-2B cell line comparing with that of LNCaP cells^26^. Next, we investigated whether CUL4B correlated to CRPC. As shown in Figure S6c, CUL4B expression is much higher in C4-2B and LNCaP-AI cell line than that of LNCaP cells. Inhibition of CUL4B attenuates C4-2B proliferation ability under androgen-deprived condition (Figure S6d).

### CUL4B+/SOX4+ PCa subgroup harbors Wnt/β-catenin signaling pathway activation

GSEA revealed that DEG in patients with concurrent CUL4B+/SOX4+ expression in TCGA and GSE70769 dataset enriches for statistically significant overlapping gene signature involved in E2F, MYC and β-catenin activation (Fig. 6e). Consistent with GSEA results, ectopic expression of CUL4B with increased SOX4 expression promotes β-catenin accumulation and translocation to nucleus (Fig. 6f). Using GSEA and GO annotation analysis, we demonstrated that Wnt signaling pathway was enriched in CUL4B+/SOX4+ subgroup compared with CUL4B−/SOX4− group in the TCGA dataset (Figure S6e and S6f). Together with western blot analysis of Wnt target genes, C-MYC and Cyclin D1 (Figure S6g), our data suggested that the Wnt signaling pathway might be active in CUL4B+/SOX4+ PCa patients. Block the CUL4B-SOX4 circuit by SOX4 inhibition impairs Wnt signaling pathway activation (Figure S6h).

LGK974 is a small molecular inhibitor of Wnt pathway^27^. As shown in Figure S6i, LGK974 treatment partially blocked C-MYC and Cyclin D1 expression that were induced by CUL4B. Furthermore, LGK974 treatment partially blocked CUL4B-induced cell migration and proliferation in vitro (Fig. 6g–k) and in vivo (Fig. 6i, j) Therefore, our data demonstrate that LGK974 treatment could partially block PCa cell transformation and cell motility caused by CUL4B expression.

## Discussion

This is the first study to systematically characterize the role of CUL4B in PCa progression. CUL4B may act as an oncogenic in PCa cells, evidenced by induction of cell proliferation, invasion, and EMT program. We showed that CUL4B overexpression was correlated with high Gleason score and unfavorable prognosis. Previously, we and others have shown that CUL4B expression is oncogenic in cancer of liver^12^, esophagus^8^, cervix^11^, and cholangio carcinoma^28^. However, Qian et al. also demonstrated that CUL4B could negatively regulate a cancer-supporting microenvironment in hematopoietic system^29^. Thus, the function of CUL4B in cancer development and progression is dichotomous, which might be in part due to organ-specific actions and the different cellular contexts of tumors.

EMT is executed by EMT-activating transcription factors (EMT-TFs), including SNAIL, TWIST and ZEB families. EMT-TFs contributes to tumor growth, metastasis, as well as resistant to therapy. Cancer cells in a tumor undergo an incomplete or partial EMT, progressing to mesenchymal state from epithelial state^30^. Dysregulated CUL4B failed to induce completely EMT, yet favoring progression to the mesenchymal state. Many investigations have demonstrated that activation or suppression of any single EMT‑TF is sufficient to induce an incomplete or partial EMT program without any compensation by other EMT‑TFs^31–33^. EMT can be induced by activation of EMT-TFs that belong to different gene families. SOX4 was identified as a master of EMT by manipulating EZH2-dependent epigenetic reprogramming, Wnt signaling and TGF-β signaling^13,34,35^. In addition to transcriptional activation of ZEB1, TWIST1, SNAIL2, SOX4 was shown to directly regulate CDH2 expression^18,36–38^. The SOX4 transcription factor is overexpressed in many types of human cancers and has been recognized as one of the 64 “cancer signature” genes, suggesting a key role in tumor progression^39^. Previously, we and others have suggested that SOX4 overexpression was correlated with poor prognosis and tumor progression through the induction of EMT and metastasis in PCa^15,34,40,41^. In this study, we demonstrated that CUL4B promotes expression of SOX4 at a posttranscriptional level, that is, via downregulation of miR-204. CUL4B has been reported to be able to repress tumor suppressors including PTEN, P16, catalyzing H2AK119 monoubiquitination that coordinates/facilitates PRC2-catalyzed H3K27me3 (ref. ^8^). Accordingly, we demonstrated that ablation of CUL4B resulted in loss of not only H2AK119 monoubiquitination but also H3K27 trimethylation catalyzed by PRC2, leading to the derepression of miR-204 level. We have thereby identified a regulatory link between CUL4B and miR-204 that is indirectly orchestrated by PRC2. Similar regulatory paradigms have been demonstrated for other cell types other than PCa^42^.

In an attempt to characterize the molecular mechanism underlying CUL4B overexpression in PCa, we revealed that SOX4 transcriptionally regulated CUL4B expression via binding to its promoter and enhanced CUL4B expression through a regulatory feedback circuit. In addition, EZH2, the core component of PRC2 involved in the regulatory of CUL4B to SOX4, is also transcriptional activated by SOX4 (ref. ^34^). These findings establish a positive feedback loop of CUL4B and SOX4 in PCa (Fig. 6k). CUL4B and SOX4 may cooperate to promote PCa progression and this cooperation might be related to resistance to androgen-deprivation treatment. This concept was supported by the clinical data that the subset of CUL4B+/SOX4+ PCa patients demonstrate poor survival and aggressive behavior, including occurrence of CRPC.

Of note, previous studies have demonstrated that both CUL4B and SOX4 could activate Wnt/β-catenin signaling in a variety of malignancies^12,40,43,44^. Yuan et al. showed that CUL4B positively activates Wnt/β-catenin pathway through transcriptionally repressing Wnt signaling antagonists and preventing GSK3-mediated β-catenin degradation^12^. SOX4 has been demonstrated to potentiate canonical Wnt signaling through a variety of mechanisms mainly through stabilization of β-catenin^40,45,46^. In this study, we have shown that CUL4B+/SOX4 PCa harbor activation of β-catenin and Wnt signaling pathway. The CUL4B-SOX4 circuit promoted β-catenin accumulation and translocation to nucleus and then Wnt signaling pathway consequentially. The Wnt signaling pathway contributes significantly to CUL4B+/SOX4 PCa progression. Wnt synthesis inhibitors blocked the tumor growth with concurrent overexpression of CUL4B and SOX4 in vivo. The subset of PCa patients with high CUL4B levels may be responsive to Wnt synthesis inhibitors or other Wnt pathway antagonists that are now entering the clinic.

Wnt pathway plays complex roles in CRPC. Studies also identified the Wnt pathway as one of the top signaling pathways with significant alterations in CRPC^47^. Considering extensive effects of Wnt/β-catenin signaling in PCa progression, inhibition of Wnt/β-catenin signaling benefits not only androgen-independent PCa, but also androgen-dependent PCa. The value of high expression of CUL4B or activation of CUL4B-SOX4 circuit in PCa risk stratification and management merits further investigation.

## Conclusions

In summary, we showed CUL4B as a key modulator of aggressive PCa by a positive feedback loop that interacts with SOX4. This regulatory circuit may have a crucial role in PCa progression. Our study may provide a novel direction for risk stratification and the clinical management of PCa.

## Materials and methods

### Tissues and tissue microarrays

A total of three tissue microarrays were constructed for 200 PCa cases using 1.0 mm cores as previously described^48^. Morphology was validated by two pathologists (B.H. and X.L.). Detailed clinical and pathological profiles were obtained from medical records and maintained on a secure relational database. This study was approved by Shandong University Medical Research Ethics Committee according to the Declaration of Helsinki. Written informed consents were obtained from all patients to approve the use of their tissues for research purposes.

### Immunohistochemistry (IHC)

IHC was performed as previously described^49^. The slides were incubated overnight with rabbit polyclonal anti-CUL4B antibody (1:500, cat no. C995; Sigma, MO, USA) and rabbit polyclonal anti-SOX4 antibody (1:100; cat no. ab80261; Abcam, Hong Kong, China). The slides were evaluated blindly by two independent pathologists (B.H. and X.L.), based on previously described scoring system. The CUL4B expressions were quantified using a four-value score for intensity (0 = negative, 1 = light, 2 = moderate, and 3 = intense) and percentage of the extent of reactivity (0 = <10%, 1 = 10–29%, 2 = 30–59%, and 3 > 60% positive cells). An immunohistochemical expression score was obtained by multiplying the intensity and reactivity extension values (range, 0–9).

### Cell lines and transfection

Human PCa cell lines (VCaP, PC3, 22RV1, DU145 and LNCaP), human prostate epithelial cell line (RWPE) and 293T (CRL-3216) were obtained from the American Type Culture Collection (Rockville, MD, USA) between 2012 and 2015 and authenticated again by short tandem repeat analysis again before and after our study. The cumulative culture length of the cells between thawing and use in this study was less than 15 passages. All of the newly revived cells were tested free of mycoplasma contamination by Hoechst 33258 staining (Beyotime, Jiangsu, China). Cells were transfected with miR-204 mimic/inhibitor, SOX4 siRNA (GenePharma, Shanghai, China), CUL4B siRNA 1#, 2# (Qiagen, Hilden, Germany), EZH2 siRNA (GenePharma, Shanghai, China) or the respective negative controls, using Hiperfect transfection reagent (Qiagen). CUL4B Plasmids were transfected with Lipofectamine 2000 (Invitrogen, Carlsbad, USA) according to the manufacturer’s instructions. For stable knockdown or overexpression of CUL4B, Lenti-shCUL4B-GFP, Lenti-CUL4B-Flag as well as their controls (Lenti-vector control and Lenti-shSCR) were transfected into VCaP or DU145 cells. The overexpression sequences and targeted sequences for siRNAs and shRNAs were described in Supplementary Table 2.

### In vitro proliferation, migration and invasion assays

After transfection with the indicated miRNA mimics or inhibitor, siRNA or plasmids, wound-healing, migration and invasion assays were performed according to the protocols as previously described^49^. Cell proliferation was measured by EdU assays (Ribobio, Guangzhou, China), 3-(4,5-dimethylthiazol-2-yl)-5-(3-carboxymethoxyphenyl)-2-(4- sulfophenyl)- 2H-tetrazolium (MTS) assays (Promega, Madison, USA) or colony formation assays according to the manufacturer’s protocol.

### RNA extraction and quantitative real-time PCR (RT-PCR)

RNA isolation and RT-PCR were performed according to the manufacturer’s instructions. Total RNA was extracted with Trizol reagents following the manufacturer’s instructions (Invitrogen). MRNA levels of primary miRNAs, SOX4, and CUL4B were assayed by SYBR Green PCR kit (Toyobo, Osaka, Japan) and mature miRNA expression levels were quantitated using MicroRNA Assay Kit (Takara, Otsu, Japan). GAPDH and U6 were used as an endogenous control for mRNA and miRNA, respectively. Primers used were described in Supplementary Table 2.

### Western blot

Western blot was performed as previously described^49^. The membranes were incubated overnight with antibodies against E-cadherin (1:1000; cat no. #3195; CST), Vimentin (1:1000; cat no. #5741S; CST), N-cadherin (1:1000; cat no. ab18203; Abcam), β-catenin (1:1000; cat no. # 8480S; CST), Cyclin D1 (1:1000; cat no. ab134175; Abcam), SOX4 (1:1000; cat no. ab80261; Abcam), CUL4B (1:2000; cat no. C995; Sigma) and GAPDH (1:1000; cat no. ab0037; Abway). Immunoreactivity was visualized using an enhanced chemiluminescence kit (Millipore, Darmstadt, Germany).

### Immunofluorescence

Immunofluorescence assay was performed as previously reported^49^. Briefly, cells were seeded on glass coverslips, fixed, then incubated with primary antibodies (SOX4, E-cadherin, N-cadherin and Vimentin, dilution 1:100; CUL4B and β-catenin dilution 1:200) serially overnight at 4 °C, followed by Alexa Fluor-594 or -488-conjugated secondary antibodies (Proteintech). Nuclei were stained with prolong gold antifade reagent with DAPI (Invitrogen).

### Bioinformatics analysis

The TCGA (The Cancer Genome Atlas, *n* = 439) dataset was downloaded from http://gdac.broadinstitute.org/. Datasets of GSE6956 (ref. ^50^), GSE68545 (ref. ^51^), GSE35988 (ref. ^52^), GSE32269 (ref. ^53^), and GSE11914(ref. ^54^) were downloaded from Gene Expression Omnibus (GEO) database (http://www.ncbi.nlm.nih.gov/geo). The expressed genes were subsequently analyzed for enrichment of biological themes using GSEA (http://www.software.broadinstitute.org/), Heatmap packages, Yyplot packages, and ClusterProfile package (R version) were utilized as previously described^55^.

### Chromatin immunoprecipitation (ChIP) assay

ChIP assay was performed according to the manufacturer’s protocol (ChIP Assay Kit, Millipore). Briefly, the cells were cross-linked with 1% formaldehyde, sheared to 200−500 bp fragments, and subsequently precipitated by anti-CUL4B (Sigma), anti-SOX4 (Novus) and anti-IgG antibody (Millipore) respectively. The purified chromatin was quantified by real-time PCR and the primers used were described in Supplementary Table 2.

### Dual luciferase assay and reporter constructs

Cells transfected with indicated plasmid and miRNA mimics/inhibitor were harvested and subjected to luciferase reporter assay using the dual luciferase assay reporter system (Promega). The wild-type and mutant SOX4 3′-UTR vector were constructed from GenePharma (Shanghai). The CUL4B promoter reporter construct was generated by cloning of the promoter region of the gene upstream from the luciferase reporter in the pGL3-basic vector (Life Technologies). Primers for PCR amplification and point mutations were introduced in Supplementary Table 2. VCaP and HEK293T cells were transfected with the reporter and indicated plasmids or microRNA mimics/inhibitors, and siRNAs. Cell lysates were harvested 48 h after transfection and subjected to luciferase reporter assay using dual-luciferase reporter assay system (Promega).

### Tumor xenografts

Male athymic nude mice (nu/nu; 4 weeks old) were purchased from Weitonglihua Biotechnology (Beijing). Nude mice (nu/nu, male 3–4 weeks old) were injected subcutaneously with 2×10^6^ VCaP stable cells. The tumor growth was monitored and measured with calipers every 3 days. For LGK974 treatment assay, DU145 cell line was infected with lentivirus containing either empty control vector (Vector) or CUL4B. The stable-transfected cells were implanted subcutaneously into the flank regions of nude mice. When a tumor became palpable, LGK974 or PBS (control) was injected intraperitoneally every 2 days for 14 days. The in vivo experiments were separated into four groups: Vector + PBS, CUL4B + PBS, Vector + LGK974, CUL4B + LGK974, *n* = 5/group). All animal experiments were conducted in strict accordance with the principles and procedures approved by the Shandong University Animal Care Committee.

### Statistical analysis

Statistical analysis was carried out using Graphpad prism 5 or SPSS 20.0 software, with *P* < 0.05 considered statistically significant. Statistical comparisons between groups were analyzed using two-sided Student’s *t* test and Mann−Whitney test. Correlation significance was assessed using *χ*^2^ test and Pearson’s correlation coefficient test.

## Supplementary information

Supplementary Figure Legend

Figure S1

Figure S2

Figure S3

Figure S4

Figure S5

Figure S6

Supplementary Table 1

Supplementary Table 2

## Data Availability

All data and computer code supporting the findings of this study are available from the authors upon request.
