# Supplementary material for: CUL4B promotes prostate cancer progression by forming positive feedback loop with SOX4
Source: Oncogenesis. 2019 Mar 14;8(3):23. doi: 10.1038/s41389-019-0131-5 (PMC6418142; doi:10.1038/s41389-019-0131-5)
Supplement: Supplementary file 1 — Supplementary Figure Legend [file 41389_2019_131_MOESM1_ESM.doc]

**Supplementary Figure Legends**

***Figure S1. Ectopic CUL4B expression promotes proliferation and invasion.***

(a) Western-blot analysis of CUL4B protein levels in human PCa cell lines.

(b) Western-blot analysis of CUL4B protein levels in VCaP and DU145 cells. VCaP cells were transient transfected with siRNA targeting CUL4B (si4B) ,negative control (NC) or stably transfected shRNA targeting CUL4B(shCUL4B) or negative control (shSCR). The CUL4B levels in these established cell lines were veriﬁed by western-blot after transfection.

(c) Validation of CUL4B over expression by western-blot in CUL4B-overexpressing and control DU145 and VCaP cells. DU145 cells were transient transfected with empty plasmids (Flagα), plasmid expressing CUL4B (Flag4B), or stably transfected empty plasmid (Vector), plasmid expressing CUL4B (CUL4B).

(d) Cell viability as assessed by MTS assay at different time points, ranging from 0 to 72 h in VCaP cells. si4B 2#/NC: CUL4B was knockdown in VCaP cells by transfection of siRNA targeting CUL4B(si4B 2#) or a negative control siRNA (NC).

(e-g) MTS (e) , colony formation assays (f), EdU assay（g）and Transwell assay (h) performed on CUL4B-overexpressing and control VCaP cells.

(i-j) Effect of CUL4B on turmorigenesis *in vivo* evaluated with xenografts model derived from DU145 cells. DU145 cells with stable expression of Vector/CUL4B subcutaneously injected into nude mice. Representative images of xenograft tumors (i), and the average weight of tumor mass in each group (j) were shown. *P < 0.05, ** P<0.01.

***Figure S2. CUL4B promotes PCa EMT in vitro.***

(a-b) The mRNA level of E-cadherin, N-cadherin and Vimentin determined by real time PCR in VCaP and DU145 cells. VCaP cells were transient transfected with siRNA targeting CUL4B (si4B 1#, si4B 2#), negative control (NC), empty plasmid (Flagα) and plasmid expressing CUL4B (Flag4B). DU145 cells were transient transfected with empty plasmid (Flagα) and plasmid expressing CUL4B (Flag4B).

(c) The expression of E-cadherin, N-cadherin, and Vimentin determined by western-blot of CUL4B-overexpressing and control VCaP cells. GAPDH was used as the loading control.

(d) Immunofluorescence analysis of expression levels of EMT related marker (E-cadherin and Vimentin) in CUL4B-overexpressing and control VCaP cells. Scale bar, 50μm.*P < 0.05, ** P<0.01.

***Figure S3. CUL4B does not regulate SOX4 expression at mRNA level***

The mRNA level of CUL4B and SOX4 were quantified by real time PCR in VCaP (a,b) and PC3 cells (c,d) transiently transfected with siRNA targeting CUL4B (si4B), negative control (NC), empty plasmid (Flagα) and plasmid expressing CUL4B (Flag4B).

***Figure S4. Screen of miRNAs involved in CUL4B-induced SOX4 expression***

(a-b) Expression of miRNAs in VCaP cells. VCaP cells were transient transfected with siRNA targeting CUL4B (si4B 1#), negative control (NC), empty plasmid (Flagα) and plasmid expressing CUL4B (Flag4B), and then the expression levels of 8 candidate miRNAs were monitored in response to CUL4B knockdown or overexpressed using real time PCR assay.

***Figure S5. Expression of miR-204 correlates with expression of short, but not long, transcripts of TRPM3***

(a) Schematic representation of indicated TRPM3 transcripts and the location of miR-204. MiR-204 locates in intron 6 and is expressed in the same orientation as TRPM3 mRNA. The long transcript, ENST00000377110, encoding the full-length protein, and short transcript, ENST00000361823, were presented separately. Specific primes used for PCR was designed to indicated location.

(b) Analysis showing correlation of miR-204 and TRPM3 expression in human PCa in TCGA datasets. Spearman r=0.439, P<0.0001.

(c) ChIP-qPCR analysis of recruitment of CUL4B and H2AK119ub1 at promoters of miR-204 in VCaP cells. Purified rabbit IgG was used as a negative control for background enrichment signal. ChIP enrichments were presented as fold over background signal. Error bars represent mean ±SD of three independent experiments.

(d) The mRNA level of VEGFA and Pre-miR-204 determined by real time PCR in DU145 cells. DU145 cells with stable expression of Vector/CUL4B was treated with Thalidomide or control.

***Figure S6. CUL4B+/SOX4+ defines a subset of PCa patients with poor prognosis and Wnt pathway activation***

(a) Correlation heatmap showing unsupervised clustering analysis of CUL4B+/SOX4+ patients and CUL4B-/SOX4- patients in TCGA dataset. The annotation color bars on the top and the side of the heatmap represent the different classes. Purple: patients with concurrent high CUL4B and SOX4 expression (CUL4B+/SOX4+); Green: patients with concurrent low CUL4B and SOX4 expression (CUL4B-/SOX4-)

(b) The correlation between CUL4B expression and overall survival in Qilu PCa cohort assessed by Kaplan-Meier survival analysis (n=200, p=0.01, Log-rank test).

(c) Western-blot analysis of CUL4B protein levels in indicated PCa cell lines.

(f) Cell viability as assessed by MTS assay in C4-2B cells. si4B 1#/NC: CUL4B was knockdown in VCaP cells by transfection of siRNA targeting CUL4B (si4B 1#) or a negative control siRNA (NC).

(e) Hierarchical clustering and heatmap of significantly differentially expressed genes between CUL4B+/SOX4+ and CUL4B-/SOX4- patients. Enriched GO terms shown in the left panel.

(f) Wnt pathway signature enriched in CUL4B+/SOX4+ subgroup compared to CUL4B-/SOX4- subgroup in TCGA dataset. Enrichment scores (ES) were shown on the y-axis. X-axis bars represent individual genes of the indicated gene sets. ES=-0.33, P=0.03, FDR *q*=0.25.

(g) Western-blot of CUL4B, c-MYC and CyclinD1 in indicated PCa cells. VCaP cells were transient transfected with siRNA targeting CUL4B (si4B) ,negative control (NC) or stably transfected shRNA targeting CUL4B (shCUL4B) or negative control (shSCR). DU145 cells were transient transfected with empty plasmid (Flagα), plasmid expressing CUL4B (Flag4B), or stably empty plasmid (Vector), plasmid expressing CUL4B (CUL4B).

(h) CUL4B induction of C-MYC and Cyclin D1 blocked by siSOX4. DU145 cells were co-transfected with Flagα/Flag4B and siSOX4/NC and analyzed by western-blot.

(i)Western-blot of CUL4B, C-MYC and Cyclin D1 in indicated PCa cells. VCaP cells with stable expression of Vector/CUL4B was treated with LGK974 (10uM) or control.

(j-k) Cell migratory (i) and proliferation (j) capacities of VCaP cells. VCaP cells with stable expression of Vector/CUL4B was treated with LGK974 (10uM) or control before MTS assay (i) and transwell migration assay (g) * P<0.05, **P<0.01, ***P<0.001
