# Supplementary figures and images for: CUL4B promotes prostate cancer progression by forming positive feedback loop with SOX4
Source: Oncogenesis. 2019 Mar 14;8(3):23. doi: 10.1038/s41389-019-0131-5 (PMC6418142; doi:10.1038/s41389-019-0131-5)

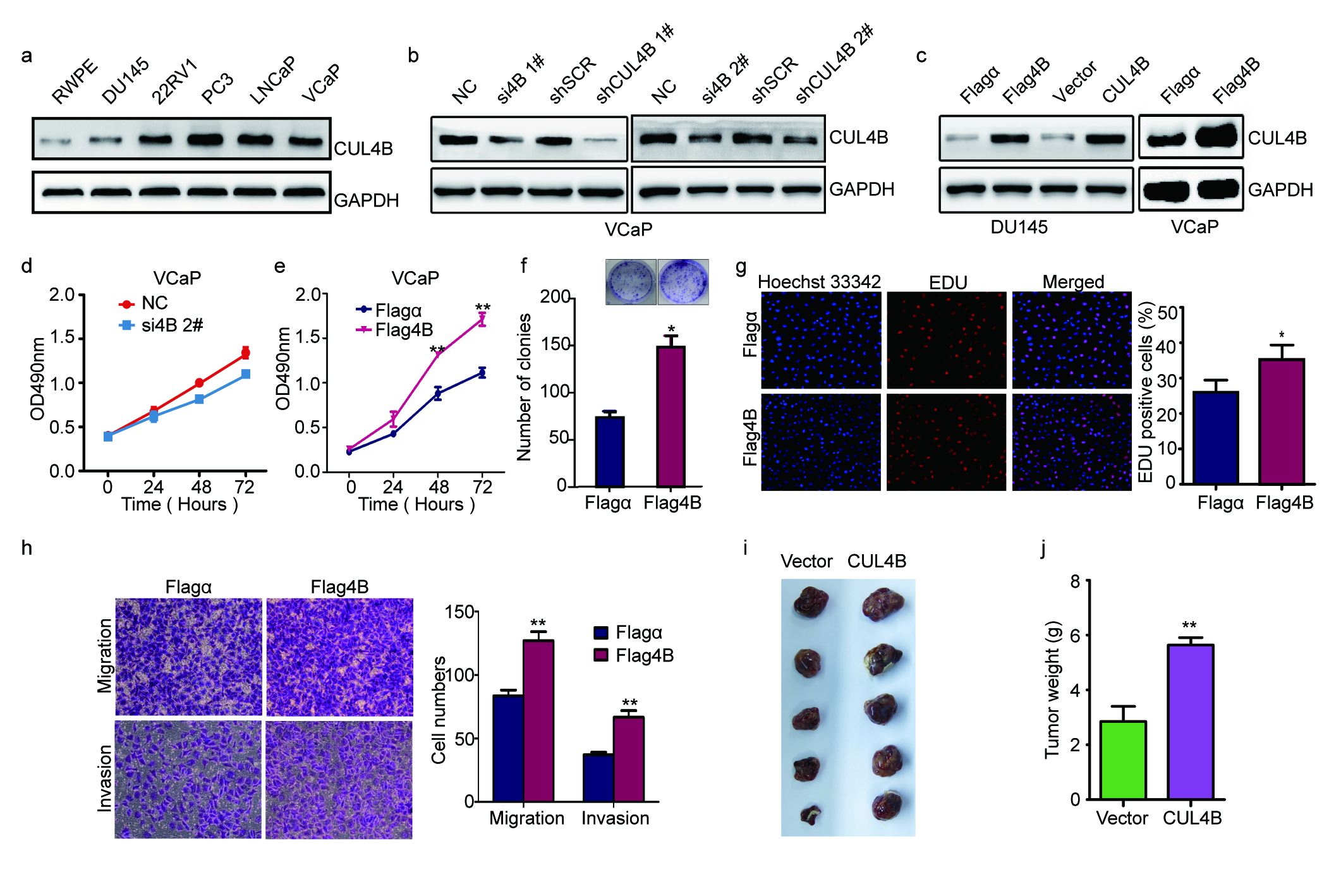

Supplement: Supplementary file 2 — Figure S1 [file 41389_2019_131_MOESM2_ESM.tif]

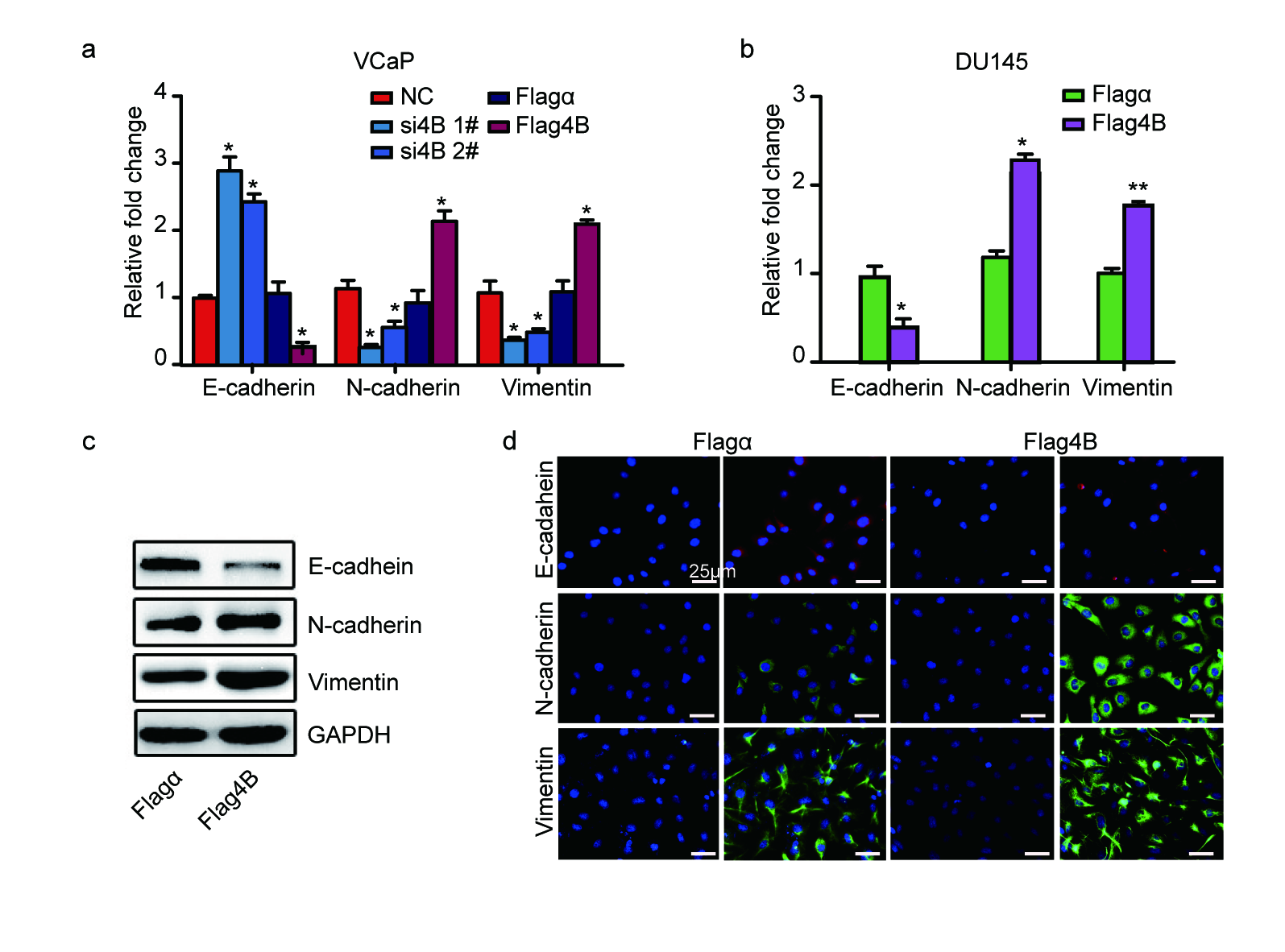

Supplement: Supplementary file 3 — Figure S2 [file 41389_2019_131_MOESM3_ESM.tif]

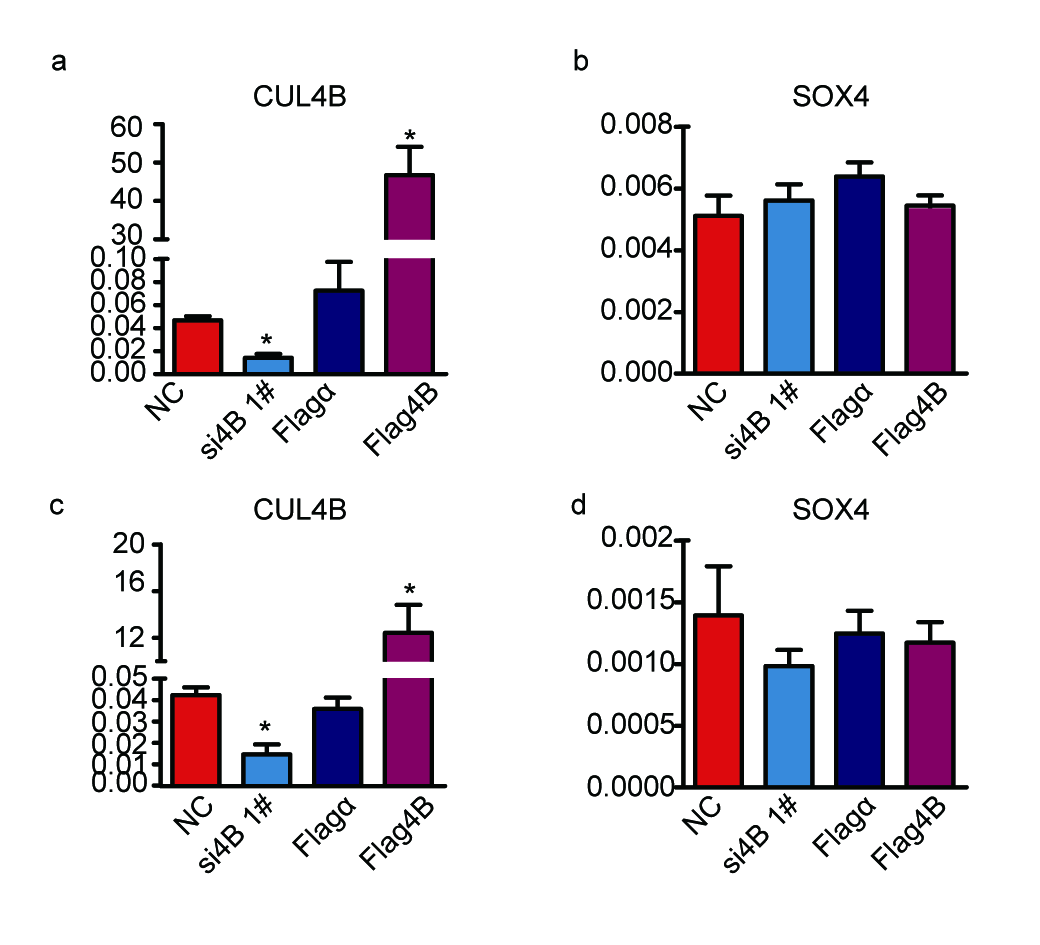

Supplement: Supplementary file 4 — Figure S3 [file 41389_2019_131_MOESM4_ESM.tif]

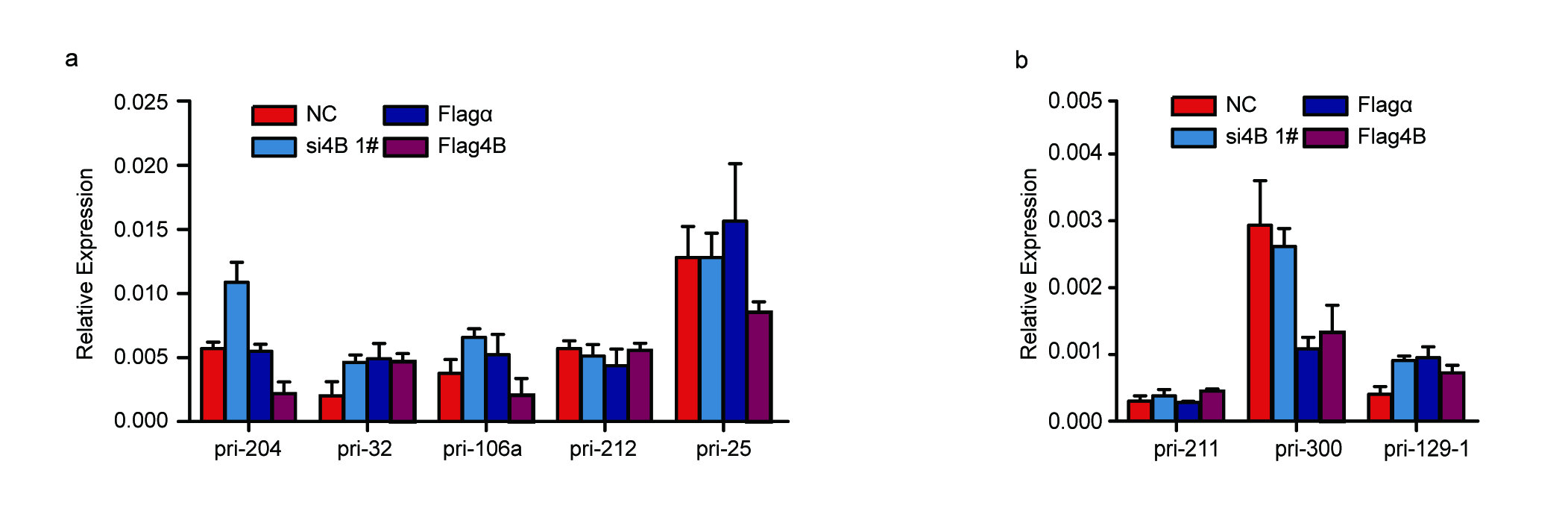

Supplement: Supplementary file 5 — Figure S4 [file 41389_2019_131_MOESM5_ESM.tif]

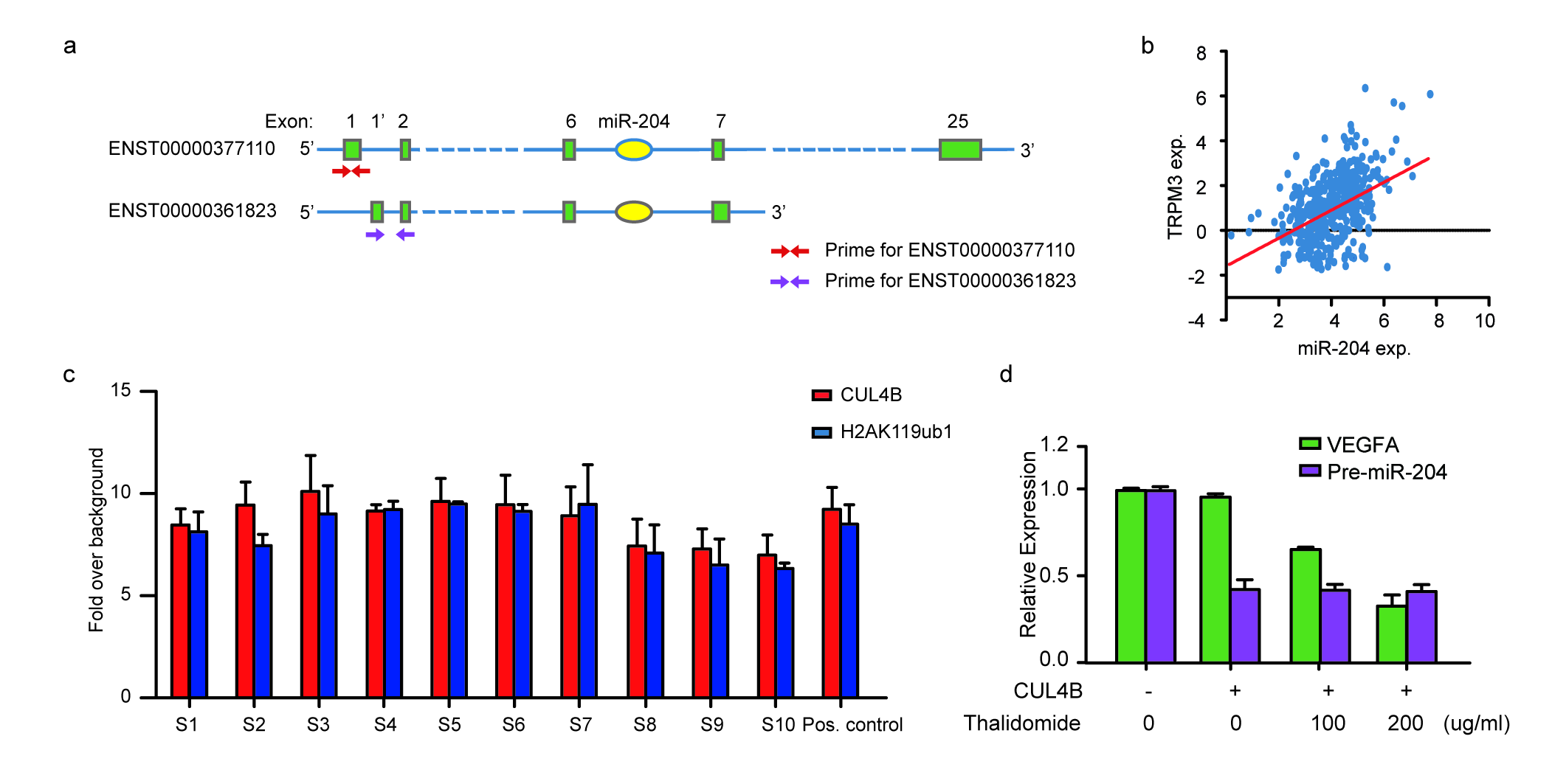

Supplement: Supplementary file 6 — Figure S5 [file 41389_2019_131_MOESM6_ESM.tif]

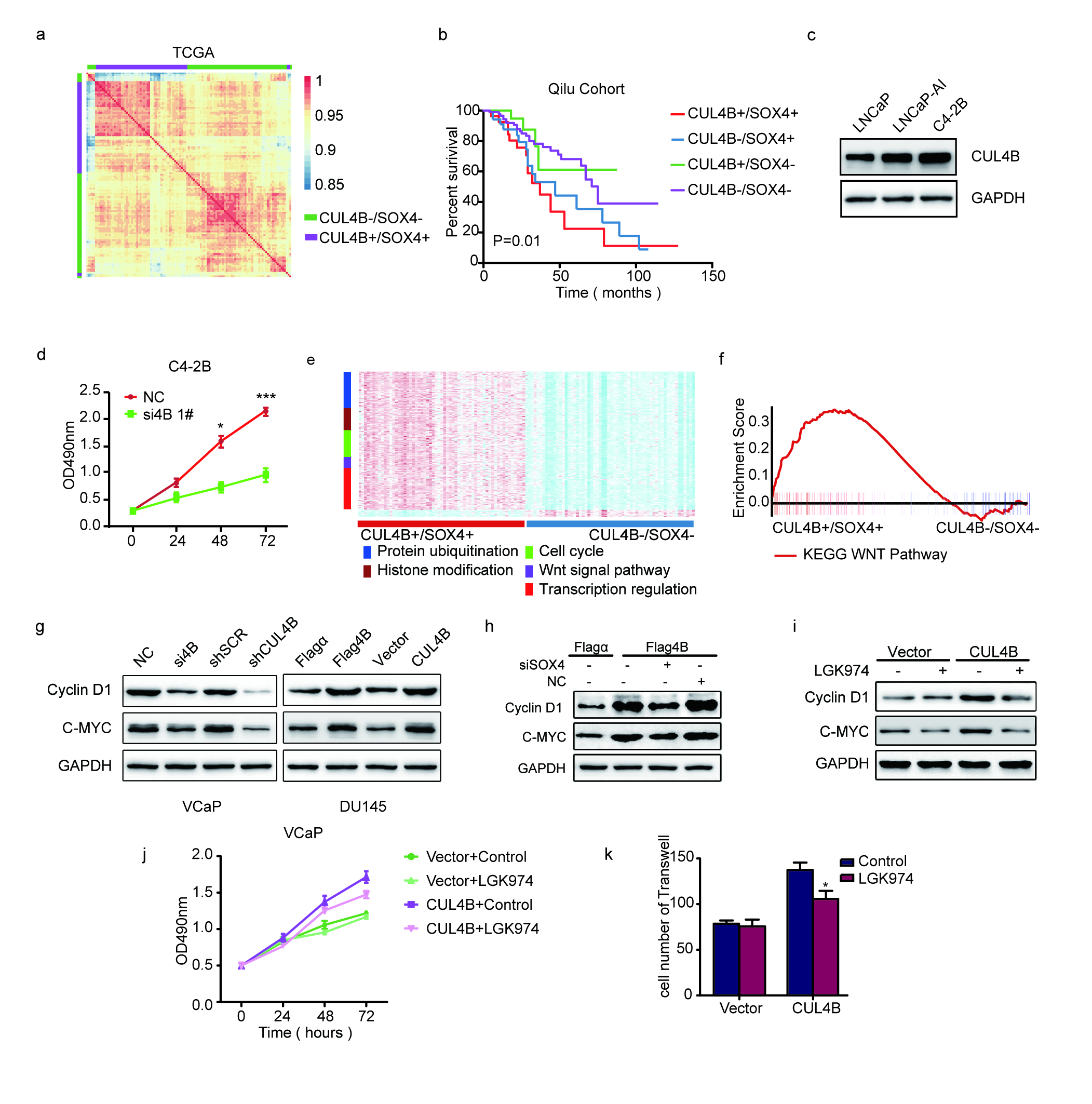

Supplement: Supplementary file 7 — Figure S6 [file 41389_2019_131_MOESM7_ESM.tif]
